# Effects of aging on brain networks during swallowing: general linear model and independent component analyses

**DOI:** 10.1038/s41598-020-79782-1

**Published:** 2021-01-13

**Authors:** Woo-Suk Tae, Sekwang Lee, Sunyoung Choi, Sung-Bom Pyun

**Affiliations:** 1 Brain Convergence Research Center, Korea University College of Medicine, Seoul, Republic of Korea; 2 Department of Biomedical Sciences, Korea University College of Medicine, Seoul, Republic of Korea; 3 Clinical Research Division, Korea Institute of Oriental Medicine, Daejeon, Republic of Korea; 4 Department of Physical Medicine and Rehabilitation, Anam Hospital, Korea University College of Medicine, Seoul, Republic of Korea

**Keywords:** Neural ageing, Ageing, Gastrointestinal system

## Abstract

Swallowing disorders occur more frequently in older adults. However, the effects of the aging process on neural activation when swallowing are unclear. We aimed to identify neural regions activated during swallowing and evaluate changes in neural activation and neural networks with aging. Using a general linear model (GLM) and independent component (IC) analyses, blood oxygen level-dependent (BOLD) signals were observed in the lateral precentral gyrus, postcentral gyrus, anterior insular cortices, supramarginal gyri, and medial frontal gyrus during swallowing. The right thalamus and anterior cingulate gyri were found to be active areas by GLM and IC analyses, respectively. In the correlational analyses, age was negatively correlated with BOLD signals of the lateral precentral gyri, postcentral gyri, and insular cortices in swallowing tasks. Additionally, correlation analyses between ICs of all participants and age revealed negative correlations in the right supramarginal gyrus, both anterior cingulate cortices, putamen, and cerebellum. In the network analysis, the BOLD signal positively correlated with age in the default mode network (DMN), and was negatively correlated in the lateral precentral gyri, postcentral gyri, and insular cortices. The amplitude of low-frequency fluctuations was significantly decreased in the DMN and increased in swallowing-related areas during swallowing tasks. These results suggest that aging has negative effects on the activation of swallowing-related regions and task-induced deactivation of the DMN. These changes may be used to detect early functional decline during swallowing.

## Introduction

Swallowing is the process of sending food through the mouth and pharynx to the esophagus. Swallowing disorders (dysphagia) are experienced by about 4% of adults each year, and are more common in older adults, affecting approximately 37.6% of adults older than 65 years living in the community^1,2^. This difficulty in swallowing is the most important risk factor for pneumonia in elderly residents in long-term care facilities^3^. The primary cause of swallowing dysfunction in older adults is an age-related disease, leading to oropharyngeal or esophageal dysfunction^4^. However, even in healthy elderly people without any specific disease, swallowing may be impaired by sensorimotor changes caused by aging^5^. The loss of muscle strength and the loss of taste and smell due to aging have been reported to worsen swallowing performance^6,7^.

Recent advances in functional magnetic resonance imaging (MRI) techniques have provided insight into the brain structures involved in swallowing and how neural activation changes due to the effects of aging. For example, fMRI studies have shown that cortical activity related to swallowing is not only limited to the motor domain but is a complex process that encompasses sensory and cognitive aspects as well^8,9^. In addition, it has been reported neural processing related to swallowing changes due to aging^5,10^. Amplitude of low-frequency fluctuations (ALFF) analysis allows for the evaluation of regional neural activity based on measurement of the blood oxygen level-dependent (BOLD) signal in the low-frequency range^11^. However, it remains unclear whether neural activation during swallowing increases or decreases with increasing age. In a previous study, BOLD activity related to swallowing was reported to increase due to aging, and this was explained as a compensatory mechanism^10^. Conversely, some studies reported a decrease in somatosensory activation due to aging^12^. Humbert et al. reported that BOLD activation on the right side of the pre- and postcentral gyri was higher in the older group than in the younger group, while activation on the left side was lower^13^. These contradictory results reveal that further research is needed on the effects of the aging process on neural activation during swallowing.

The default mode network (DMN) is an area of the brain that is activated in the resting-state and shows deactivation when attempting to perform a specific task^14^. The topography of the DMN can be obtained from resting-state fMRI^15^ or task-based fMRI^16^. These two methods reflect overlapping neurophysiological processes, but it has recently been reported that negative BOLD responses obtained from task-based fMRI are more correlated with task performance than resting-state fMRI^17^. Many researchers have reported that task-induced deactivation (TID) of the DMN decreases during the aging process^18,19^. However, it is not known how aging affects BOLD signal changes in the DMN when performing swallowing tasks. The present study applied a pluralistic approach using a general linear model (GLM) and an independent component analysis (ICA) model to identify changes in brain neural networks with healthy aging. This approach is known to help increase the reliability of the results and reveal results that are not identified in other methods^20^. The GLM applies a model-based approach using a canonical hemodynamic response function, while ICA is suited for functionally classifying brain regions by applying a data-driven approach^21,22^. We hypothesized that changes in neural activation and functional connectivity during swallowing will be affected by aging. The purpose of this study was to identify activated neural regions involved in swallowing and to evaluate changes in neural activation and neural networks with aging.

## Results

Forty-six healthy individuals were recruited in this study (45.7 ± 18.37 years, range 19–73 years; 19 male and 27 female). The age distribution was as follows: 1 participant aged 19 years, 11 in their 20 s, 13 in their 30 s, 5 in their 40 s, 0 in their 50 s, 8 in their 60 s, and 8 in their 70 s (Supplementary Fig. 1 online). None of the participants met the exclusion criteria; therefore, all 46 participants performed the fMRI experiment. In fMRI acquisition, the head motions of all participants were within 1 voxel size (isotropic 3 mm) in translation and 2° in rotation.

**Figure 1:**
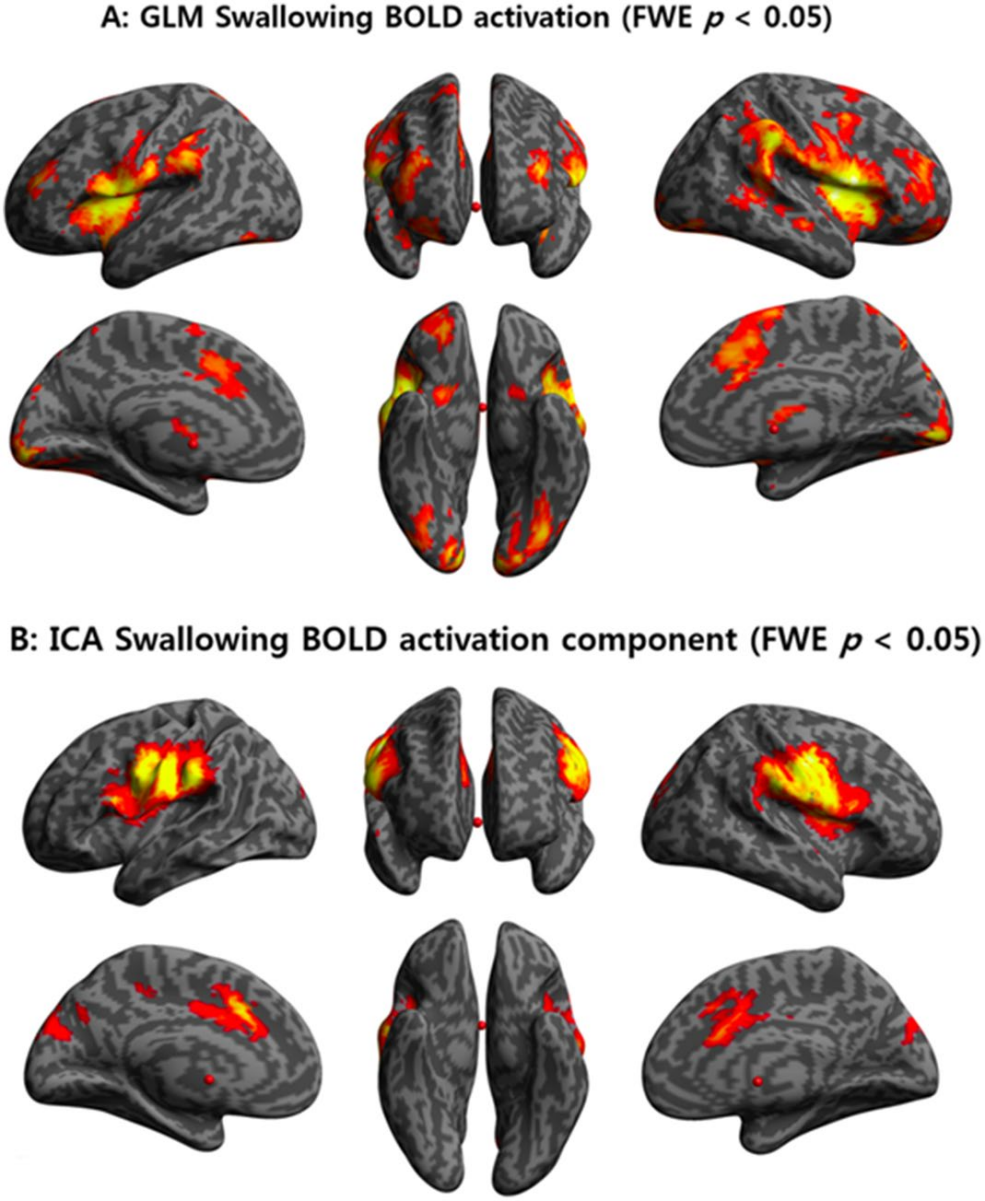
BOLD signal in swallowing tasks in general linear model and independent component analyses. (**A**) In GLM analyses, significant BOLD signals were seen in both the lateral precentral gyri, postcentral gyri, anterior insular cortices, supramarginal gyri, pre-SMA, thalami, and visual cortices. (**B**) IC 17 showed the strongest positive correlation with BOLD signal (r = 0.64, *p* < 0.001) in the swallowing task in both the lateral precentral gyri, postcentral gyri, anterior insular cortices, supramarginal gyri, pre-SMA, and anterior cingulate gyri. GLM, general linear model; BOLD, blood oxygen level-dependent; SMA, supplementary motor area; IC, independent component; FWE, family-wise error.

### GLM analyses

**Figure 2:**
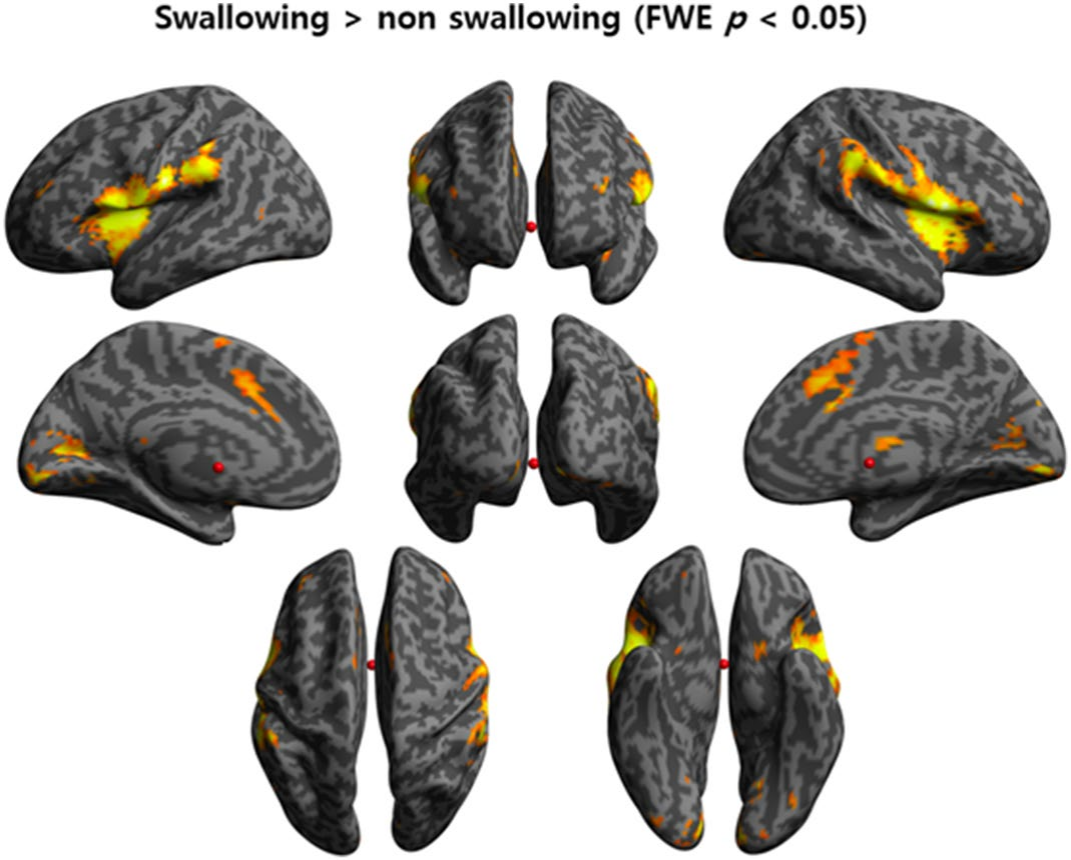
BOLD signal in swallowing task contrasted with that in non-swallowing task. In the cognition-subtracted activation of swallowing versus non-swallowing tasks, the BOLD signal is predominantly seen in both the lateral precentral gyri, postcentral gyri, anterior insular cortices, supramarginal gyri, pre-SMA, and right thalamus. BOLD, blood oxygen level-dependent; SMA, supplementary motor area; FWE, family-wise error.

During swallowing tasks, significant BOLD activation was observed in both the inferior pericentral gyri, supramarginal gyri, anterior insular cortices, pre-supplementary motor area (pre-SMA), and visual cortices in the GLM analysis (Fig. 1A). The BOLD activation in the swallowing task contrasted with that in the non-swallowing task revealed dominant activity in the areas of both the lateral precentral gyri, postcentral gyri, supramarginal gyri, pre-SMA, and right thalamus, and showed a more spatially localized pattern than that in the swallowing task without contrast (Fig. 2).

**Figure 3:**
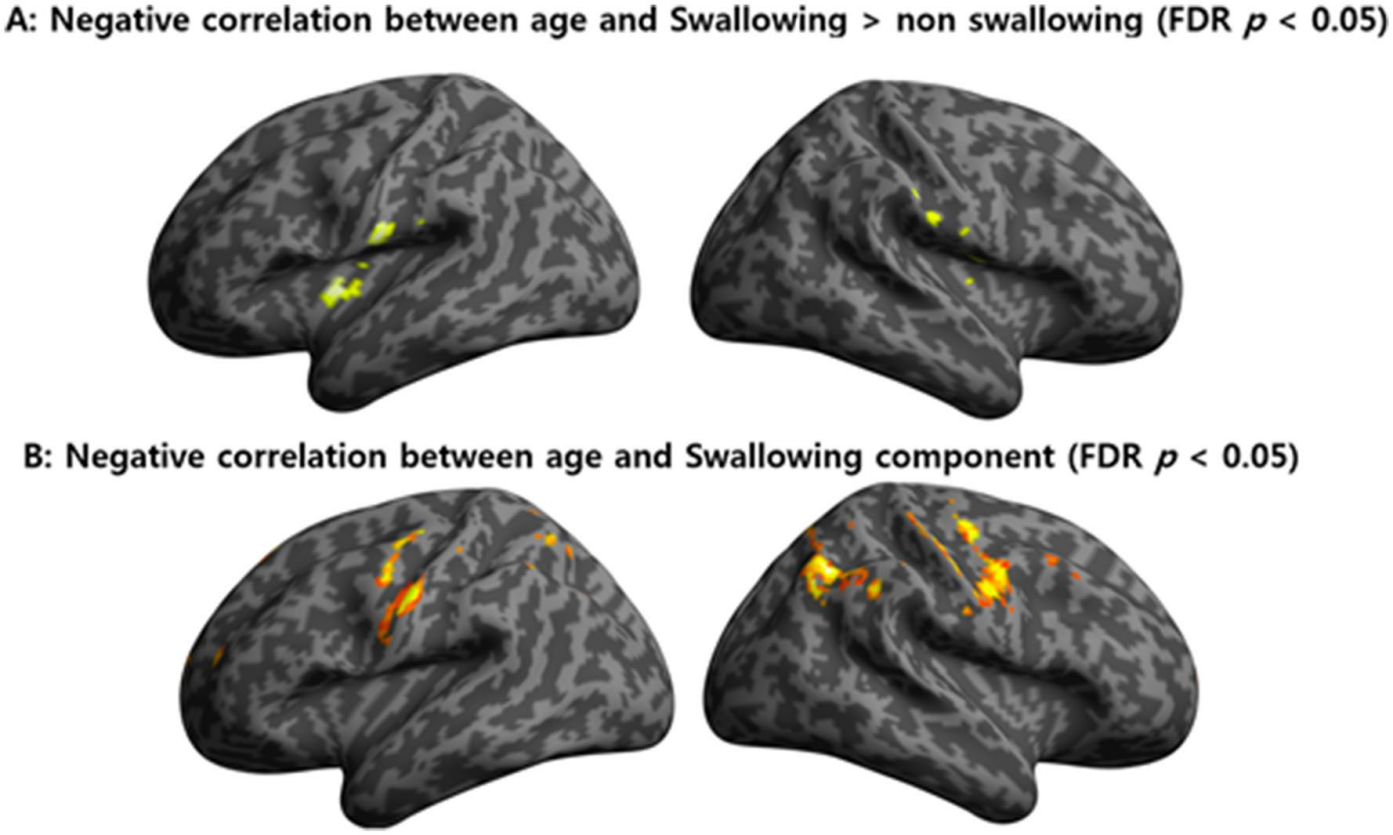
Negative correlation between age and BOLD signal during swallowing task. In the GLM, cognitively contrasted BOLD signals between swallowing and non-swallowing tasks showed a decline with healthy aging in both the lateral precentral gyri, postcentral gyri, and insular cortices (**A**) (r = 0.56, *p* < 0.001). Independent component 17, which was most positively correlated with BOLD signal (r = 0.64, *p* < 0.001) in the swallowing task, showed a clear negative correlation with age in both the lateral precentral gyri, postcentral gyri, and right supramarginal gyrus (**B**). BOLD, blood oxygen level-dependent; GLM, general linear model; FDR, false discovery rate.

The correlation between age and BOLD signal in the swallowing task was not significant (false discovery rate [FDR] *p* > 0.05). However, BOLD activation in the swallowing task contrasted with that in the non-swallowing task negatively correlated (r = 0.56, *p* < 0.001) with age in the lateral precentral gyrus, postcentral gyrus, and insular cortex (Fig. 3A).

### Multivariate analyses

Among the 26 independent components (ICs), 12 were positively correlated with the swallowing task. IC 17 had the strongest positive correlation with BOLD activation (*r* = 0.64, *p* < 0.001) during the swallowing task in both the lateral precentral gyri, postcentral gyri, supramarginal gyri, anterior insular cortices, pre-SMA, and anterior cingulate gyri (Fig. 1B).

Correlation analyses between all ICs and age revealed a negative correlation in both the lateral precentral gyri, postcentral gyri, and right supramarginal gyrus (Fig. 3B, IC 17), and the anterior cingulate cortex (ACC) (IC 12), putamen (IC 15), and cerebellum (IC 18) (FDR *p* < 0.05).

### ALFF analysis

**Figure 4:**
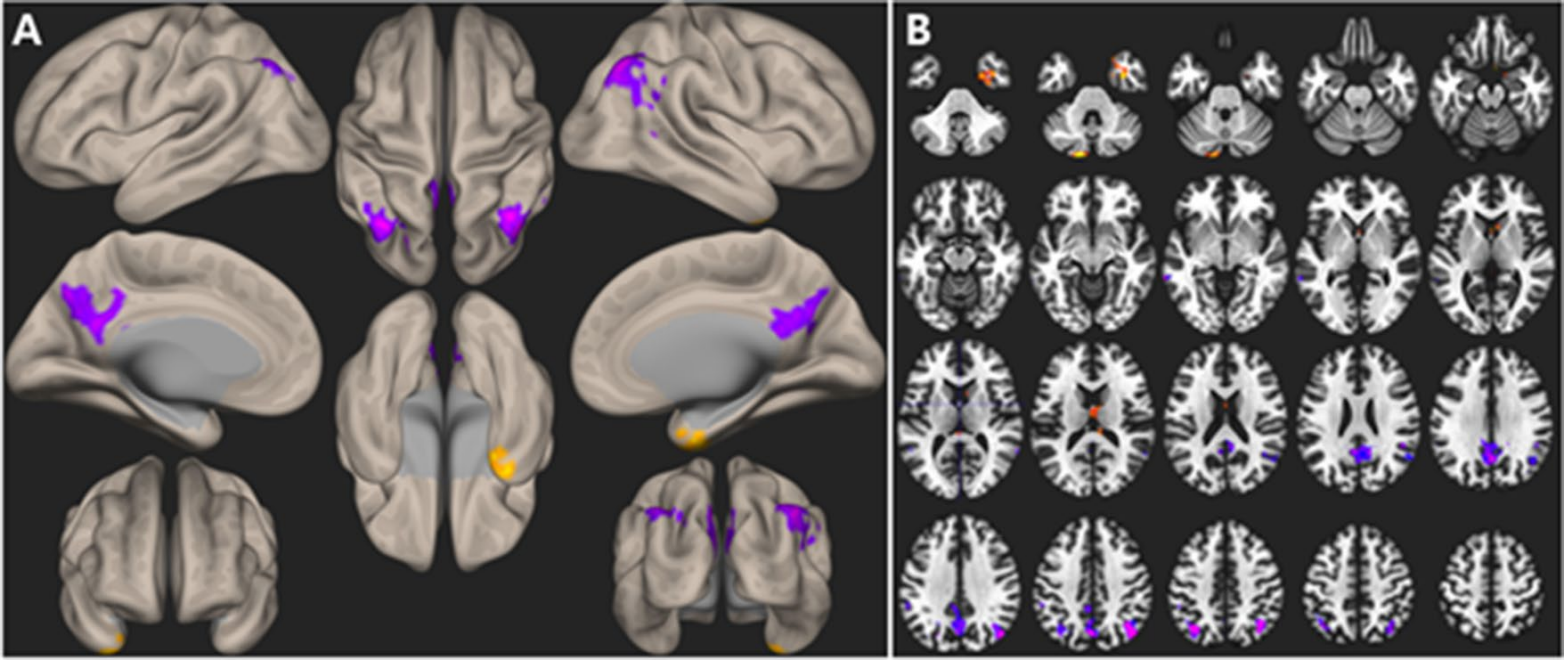
ALFF results. In the DMN area, ALFF was significantly lower during swallowing tasks than during non-swallowing tasks. In contrast, ALFF in the medial thalamus, right anteromesial temporal lobe, and left inferior semilunar lobule of the cerebellum were significantly higher. Statistical significance was set at FDR-corrected *p* < 0.05. ALFF, amplitude of low-frequency fluctuations; DMN, default mode network; FDR, false discovery rate.

ALFF in the DMN area was significantly lower during the swallowing tasks than during the non-swallowing tasks. In contrast, ALFF in the medial thalamus, right anteromesial temporal lobe, and left inferior semilunar lobule of the cerebellum were significantly higher (FDR *p* < 0.05, Fig. 4).

### Network analyses

**Figure 5:**
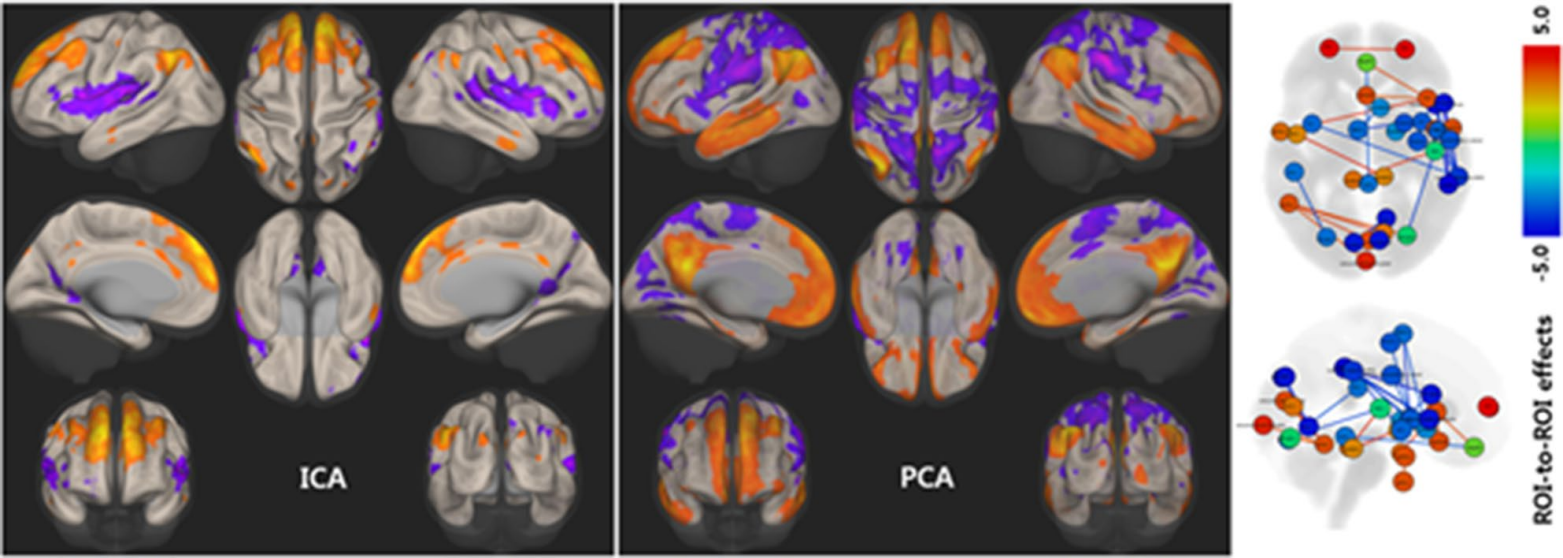
Network analysis of age and BOLD signal change during swallowing. During the 12-min tasks, the BOLD signals of the DMN areas were more positively correlated with age, whereas the BOLD signals of both lateral precentral gyri, postcentral gyri, and insular cortices declined with normal aging. The BOLD signal changes in the ICA and PCA showed similar patterns, although PCA more clearly demonstrated involvement of the DMN. ROI-based network analysis (right panel) suggests age-related decline of the BOLD signal in motor and insular areas, but strengthened networks in DMN areas such as the medial prefrontal, posterior cingulate cortices, dorsal superior frontal, angular gyri, and temporal pole. The color bar depicts t-values. Statistical significance was set at FDR-corrected *p* < 0.05. BOLD, blood oxygen level-dependent; DMN, default mode network; ICA, independent component analysis; PCA, principal component analysis; ROI, region of interest; FDR, false discovery rate.

During swallowing tasks, the BOLD signal showed a positive correlation with age in DMN areas. In contrast, the BOLD signal in the lateral precentral gyri, postcentral gyri, and insular cortices were negatively correlated with age. ICA and principal component analysis (PCA) of the BOLD signal change revealed similar patterns, but PCA demonstrated clearer involvement of the DMN. Region of interest (ROI)-based network analysis suggested an age-related decline in the BOLD signal in motor and insular areas and strengthened networks in DMN-related areas such as the medial prefrontal cortex, posterior cingulate cortices, dorsal superior frontal, angular gyri, and temporal pole (FDR *p* < 0.05, Fig. 5).

## Discussion

In this study, we evaluated the activation of neural regions during swallowing and the correlation between activation and age. Furthermore, we examined the change in BOLD signals with aging within DMN areas. The results revealed that BOLD activity during swallowing was negatively correlated with age. Moreover, network analysis showed strengthened networks in DMN areas as well as less cortical activation in motor and insular areas in older participants.

In both the GLM and ICA analyses, BOLD signal activation was observed in the primary motor and sensory areas (lateral precentral gyrus and postcentral gyrus), anterior insular cortices, supramarginal gyri, and pre-SMA (medial frontal gyrus) during swallowing. In addition, the right thalamus was found to be an active area by GLM analysis and the anterior cingulate gyri by ICA analysis. The results of our study are similar to those of previous studies. A systematic review of fMRI studies on swallowing reported that primary motor and sensory areas were the most consistently activated areas during swallowing, and activation of the anterior cingulate cortex and insular cortex was also prevalent^23^. Although these researchers reported less agreement on the activation of the supramarginal gyri, SMA, and thalamus during swallowing, other studies reported activation in all three areas during swallowing^24^. Our results show that the areas of the brain that were reported to be activated during swallowing were also effectively activated in our experimental design while lying down and swallowing saliva, increasing the reliability of the experiment.

Our results show a negative correlation between age and BOLD activation in the lateral precentral gyrus, postcentral gyrus, insular cortex, and supramarginal gyrus, which are involved in sensorimotor function^25–28^. The loss of strength and range of motion, sensory changes, and slowing of food transit, as well as age-related disease, are known to contribute to the decline of swallowing function in the elderly individuals^29^. The lateral precentral gyrus is involved in oral and facial control and starts a series of swallowing processes, and the postcentral gyrus is involved in oropharyngeal sensory processing^9^. Meanwhile, the insular cortex and supramarginal gyrus are known to be involved in sensorimotor integration. A recent study reported that this integrative functional impairment was associated with an increase in the presence of penetration/aspiration^30^. In particular, damage to the anterior insula has been reported to inhibit pharyngeal swallowing by delaying the onset of swallowing after gustatory input^31^. In summary, our results suggest that aging affects key brain structures related to sensorimotor function involved in a series of swallowing processes.

Another significant aspect of our study is that a change in the DMN with age was confirmed. The ALFF in the DMN area decreased but increased in the thalamus, right anteromesial temporal lobe, and left cerebellum, the areas related to swallowing^24,32,33^. In the network analysis, the BOLD signal decreased in the swallowing-related areas with age, while it increased in the DMN areas. Healthy aging is reported to have an effect on DMN integrity in the resting-state and TID, but this is not clearly understood^34^. Although decreased activation of the DMN with increased age has been reported^35^, an increase in the neural activity of anterior brain areas has also been reported^36^. The posterior anterior shift in aging (PASA) model suggests that the DMN shows increased deactivation in the bilateral frontal area to compensate for reduced deactivation in the temporo-occipital area^37^. In our study, the activation of the swallowing-related areas decreased and activation in the DMN was increased during aging in healthy participants. This result reflects ineffective TID of the DMN when the transition from “default mode” to swallowing task mode is required in old age^38^. Since this pattern was observed in healthy participants in our study, this result may have important implications for developing predictive markers of early functional decline in swallowing.

The limitation of this study is that the substance swallowed was only saliva. It is possible that the small amount of saliva swallowed in the experiment did not sufficiently mimic the thickness, texture, or amount of food or water swallowed during actual eating. The authors expect that experiments under conditions that diversify the type and amount of food will provide a more specific assessment of brain changes due to aging. Another limitation is that the swallowing function of the test participants was not assessed using a video-fluoroscopic swallowing study (VFSS). Among asymptomatic elderly patients, there are many abnormalities in VFSS relative to healthy young adults^39^. Therefore, there may be some healthy participants who have no symptoms but have reduced swallowing function. VFSS parameters (transit time, pharyngeal triggering time) would have provided more detailed physiological changes associated with changes in fMRI. Despite the above limitations, this study reminds us that changes to the aging brain should be understood in terms of alterations of the whole brain network rather than altered activation of focal regions. In addition, the results caused by aging in healthy people can be used as a reference to determine whether brain changes in elderly patients with swallowing disorders are pathologic or physiologic.

In conclusion, the aging process not only affects the activation of brain regions involved in swallowing, but also the TID of the DMN. This change in the DMN may be used to detect early functional decline during swallowing.

## Methods

### Participants

We recruited participants through recruitment posters posted at the outpatient clinic. In addition, the recruitment of participants was promoted to hospital staff and their families. Recruitment targets were adults 19 years of age or older who did not have a history of stroke or neurological disorders and did not complain of any swallowing difficulty. The exclusion criteria were as follows: (1) history of recurrent pneumonia, head and neck cancer, hypoxia, intubation or tracheostomy, and the status of anterior cervical spinal fusion, which may affect swallowing function; (2) throat clearing, coughing, breathing difficulty, increased secretion, voice changes, or reduced laryngeal elevation on the saliva swallow; (3) Mini-Mental Status Examination score less than 24 points, which is the criterion for suspecting cognitive decline^40^; and (4) inability to perform the saliva swallowing task by verbal command. The Clinical Research Ethics Committee of Korea University approved the study protocol (No. 2013AN0235), and informed consent was obtained from all individual participants included. All procedures performed during studies involving human participants were in accordance with the ethical standards of the institutional and/or national research committees and with the 1964 Declaration of Helsinki and its later amendments or comparable ethical standards.

### Volitional saliva swallowing task for fMRI

The participants performed 12-min tasks in an fMRI scanner. The controlled block design consisted of swallowing and non-swallowing task blocks and a control block. In each trial, a fixation crosshair was presented for 20 s in the control block, followed by a green or red dot for 4 s for the task blocks. Participants were asked to follow the instructions given in advance according to the color of the dot (green or red). In the green dot condition, patients were instructed to swallow their saliva once with minimal facial movement. In the red dot condition, patients were instructed to rest without any movement while the dot was presented on the screen. The green and red dots were presented alternately, starting with a green dot. We checked whether participants showed normal saliva swallowing using surface electromyography (EMG) with an electrode on the infrahyoid muscle while participants performed three saliva swallowing tasks before the fMRI acquisition. During the 12-min task, 15 swallowing and non-swallowing tasks were conducted. We monitored the swallowing behavior through a monitor that shows the participant’s face. E-Prime software (Psychology Software Tools, Pittsburgh, PA) was used to program the task.

### fMRI acquisition

MR images were acquired using a 3-T Siemens Trio whole-body MRI scanner (Siemens Medical Systems, Erlangen, Germany). T1-weighted magnetization-prepared rapid gradient echo images with 1-mm isotropic resolution, repetition time (TR) = 1900 ms, echo time (TE) = 2.52 ms, flip angle = 9°, were acquired in the sagittal orientation. Functional MRIs were obtained throughout the 12-min swallowing tasks using an echo-planar imaging (EPI) sequence with 3-mm isotropic resolution, TR = 2 s, TE = 20 ms, flip angle = 90°, interleaved ascending scan without a slice gap.

### fMRI analysis

#### Preprocessing

Preprocessing was performed using SPM12 (www.fil.ion.ucl.ac.uk/spm/software/spm12) in a MATLAB R2018b environment (MathWorks, Natick, MA). The EPIs of each participant were slice-timing corrected, realigned to the mean EPI to correct for head movement, co-registered to individual structural T1 MRI, and normalized to Montreal Neurological Institute (MNI) standard space using normalization parameters from the individual T1 MRI. To reduce signal loss, transformation matrices were estimated in each step, and spatially normalized images were generated in the final process. The normalized images were spatially smoothed with an 8-mm full width at half maximum (FWHM) Gaussian kernel.

#### GLM

In the first-level analysis, individual contrast images were computed using a GLM in SPM12. A design matrix comprising swallowing and non-swallowing conditions was defined while controlling for physiological and head movement-related artifacts using motion parameters generated in the realignment procedure. For the second-level analysis, individual contrast images, such as swallowing, non-swallowing, and swallowing versus non-swallowing contrasts, were fed into a one-sample t-test for each experimental condition. A one-sample t-test was then conducted to perform group statistical analyses (swallowing, non-swallowing, and swallowing > non-swallowing). Partial correlation analyses were performed to determine aging-related BOLD signal changes in each of the three conditions with the cofounding factor of sex. All statistical maps were thresholded at the level of family-wise error (FWE)- or FDR-corrected *p* < 0.05, with a minimum cluster size of 10 voxels.

#### Multivariate analyses: IC and network analyses (GIFT and CONN)

Independent component analysis (ICA) was performed using group ICA in fMRI Toolbox, GIFT (version 3.0b, http://mialab.mrn.org/software/gift)^41^. The pre-processed fMRI data were entered into GIFT. GIFT concatenated the individual data and computed participant-specific ICs and time courses. Using PCA, the dimensions of the individual data were reduced. The informax algorithm^42^ was applied for group ICA and estimated 26 ICs. To verify the stability of the ICs, ICASSO was applied 20 times^43^. All individual components were scaled to represent the percentage signal change. The group statistical maps of each IC were generated using a one-sample t*-*test on each particpants’s individual IC maps and then thresholded at FDR-corrected *p* < 0.05.

Functional network analyses were performed using CONN (version 2018b, https://www.nitrc.org/projects/conn); the preprocessing of fMRI data followed the standard CONN preprocessing pipeline^44^. The swallowing fMRI data were slice time-corrected and realigned to generate six rigid-body parameters to correct the participant’s brain motion. Based on realignment, fMRI data were co-registered to their 3D-T1 images, then spatially normalized to the MNI template, and spatially smoothed using an 8-mm FWHM Gaussian kernel. Using the CompCor algorithm^45^, the motion artifacts of the brain were removed. The time courses of the fMRI data were detrended, de-spiked, and filtered using a bandpass filter (0.008 Hz < f < 0.09 Hz) to decrease the effects of low-frequency signal drift.

ALFF maps measure BOLD signal power within the low-frequency band (0.01–0.10 Hz) and are used to measure spontaneous BOLD signal fluctuations in the brain. CONN’s ALFF is defined as the root mean square of the BOLD signal at each brain voxel after band- or low-pass filtering^46^.

$${\text{ALFF}}\left( x \right) = \sqrt {\frac{1}{N}\mathop \sum \limits_{t} \left( {f\left( t \right)*S\left( {x,t} \right)} \right)^{2} }$$

where ***S*** is the raw BOLD time series before band- or low-pass filtering, ***f ***is a band- or low-pass filter, and N is the number of timepoints.

CONN’s network analyses summarize the properties of the voxel-to-voxel connections in the brain into a series of reduced measures at each voxel. These processes include measures that address properties specified a priori, estimate how those properties are expressed in each participant, and then attempt to determine how those observed properties are expressed in each participant, such as group ICA and group PCA^47^. The independent and principal components of each participant were identified and correlated by the stimulus onset asynchrony of the swallowing task. The most positively and negatively correlated components of ICA and PCA were tested by partial correlation analyses with sex as a confounder.

The graph analysis from CONN is based on predefined non-directional graphs with nodes (all ROI-level of brain) and edges (supra-threshold connections). For each participant, graph adjacency matrices were calculated by thresholding the ROI to ROI correlation matrix by an absolute (e.g., z > 0.5) or relative (e.g., highest 10%) threshold. From the resulting graphs of each participant, the topological properties of each ROI of the entire network of ROIs were computed^48,49^.

## Supplementary Information

Supplementary information.

## Data Availability

The datasets generated and/or analyzed during the current study are available from the corresponding author upon reasonable request.
