# Supplementary material for: Effects of aging on brain networks during swallowing: general linear model and independent component analyses
Source: Sci Rep. 2021 Jan 13;11:1069. doi: 10.1038/s41598-020-79782-1 (PMC7806781; doi:10.1038/s41598-020-79782-1)
Supplement: Supplementary file 1 — Supplementary information. [file 41598_2020_79782_MOESM1_ESM.pdf]

Supplementary information

Title: Effects of aging on brain networks during swallowing: General linear model and independent component analyses

Authors: Woo-Suk Tae[1]*, Sekwang Lee[2]*, Sunyoung Choi[3], Sung-Bom Pyun[1,4]

Affiliations:
[1] Brain Convergence Research Center, Korea University College of Medicine, Seoul, Korea
[2] Department of Biomedical Sciences, Korea University College of Medicine, Seoul, Korea
[3] Clinical Research Division, Korea Institute of Oriental Medicine, Daejeon, Korea
[4] Department of Physical Medicine and Rehabilitation, Korea University College of Medicine, Seoul, Korea

Corresponding author:
Sung-Bom Pyun, MD, PhD
Department of Physical Medicine and Rehabilitation, Korea University Anam Hospital, Korea University College of Medicine, 73 Goryeodae-ro, Seongbuk-gu, Seoul 02841, Korea
Tel: +82-2-920-6483, Fax: +82-2-929-9951, E-mail: rmpyun@korea.ac.kr

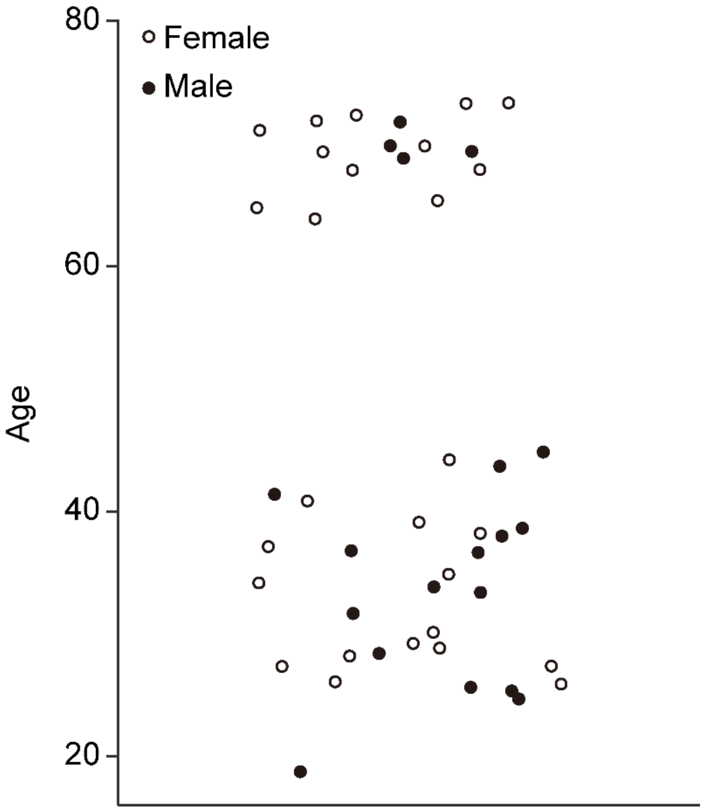

Supplementary Fig. 1 Scatter plot of sex and age distribution.
There were 46 participants in total (19 male, 27 female). The age distribution was as follows: 1 participant aged 19 years, 11 in their 20s, 13 in their 50s, 5 in their 40s, 0 in their 50s, 8 in their 60s, and 8 in their 70s.
